# Interaction of α-Melanocortin and Its Pentapeptide Antisense LVKAT: Effects on Hepatoprotection in Male CBA Mice

**DOI:** 10.3390/molecules16097331

**Published:** 2011-08-26

**Authors:** Karlo Houra, Petra Turčić, Mario Gabričević, Tin Weitner, Paško Konjevoda, Nikola Štambuk

**Affiliations:** 1 St. Catherine’s Hospital, Bračak 8, 49210 Zabok, Croatia; Email: karlo.houra@svkatarina.hr (K.H.); 2 Department of Pharmacology, Faculty of Pharmacy and Biochemistry, University of Zagreb, Domagojeva 2, 10000 Zagreb, Croatia; Email: pturcic@pharma.hr (P.T.); 3 Department of General and Inorganic Chemistry, Faculty of Pharmacy and Biochemistry, University of Zagreb, Ante Kovačića 1, 10000 Zagreb, Croatia; Email: mariog@pharma.hr (M.G.); tweitner@pharma.hr (T.W.); 4 Ruđer Bošković Institute, Bijenička cesta 54, 10002 Zagreb, Croatia; Email: pkonjev@irb.hr (P.K.)

**Keywords:** α-MSH, antisense, peptide, fluorescence, binding, hepatoprotection

## Abstract

The genetic code defines nucleotide patterns that code for individual amino acids and their complementary, *i.e.*, antisense, pairs. Peptides specified by the complementary mRNAs often bind to each other with a higher specificity and efficacy. Applications of this genetic code property in biomedicine are related to the modulation of peptide and hormone biological function, selective immunomodulation, modeling of discontinuous and linear epitopes, modeling of mimotopes, paratopes and antibody mimetics, peptide vaccine development, peptidomimetic and drug design. We have investigated sense-antisense peptide interactions and related modulation of the peptide function by modulating the effects of α-MSH on hepatoprotection with its antisense peptide LVKAT. First, transcription of complementary mRNA sequence of α-MSH in 3’→5’ direction was used to design antisense peptide to the central motif that serves as α-MSH pharmacophore for melanocortin receptors. Second, tryptophan spectrofluorometric titration was applied to evaluate the binding of α-MSH and its central pharmacophore motif to the antisense peptide, and it was concluded that this procedure represents a simple and efficient method to evaluate sense-antisense peptide interaction *in vitro*. Third, we showed that antisense peptide LVKAT abolished potent hepatoprotective effects of α-MSH *in vivo*.

## 1. Introduction

Peptides specified by the complementary RNAs often bind to each other with higher specificity and efficacy [1,2,3,4,5]. This may result from the genetic code property that codons for the hydrophilic amino acids are complemented by codons for the hydrophobic amino acids and *vice versa* [1,2,3,4,5,6,7,8,9,10,11]. The use of antisense peptides in biomedicine has been successfully applied to the modeling of more than 40 complementary peptide-receptor systems and became a valuable tool for deriving new biologically active peptides and antibodies, and performing selective peptide-receptor modulation [1,2,3,4,5,6,7,8,9,10,11]. Despite many *in vitro* studies confirming the validity of the concept, there is little evidence of direct *in vivo* modulation of the biological response to the bioactive peptide hormone using complementary peptides.

Alpha-melanotropin (α-MSH) is an ancient, evolutionally conserved, tridecapeptide derived by the proteolytic cleavage from the pro-opiomelanocortin (POMC) hormone [12,13,14]. It is currently the most widely studied melanocortin peptide in the context of tissue inflammation and cytoprotection [12,13,14]. Recently, Turčić *et al.* [15,16] showed that α-MSH exerts potent hepatoprotective effects in the mouse model of acetaminophen induced hepatotoxicity.

**Scheme 1:**
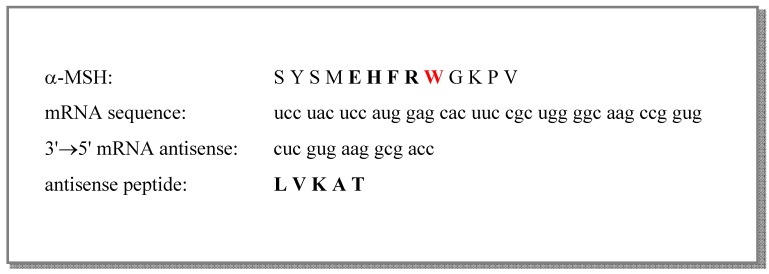
Antisense peptide to the central region of α-MSH molecule [1,2,3,4,5,6].

In this investigation we evaluated the binding of antisense peptide to α-MSH, and its effects on α-MSH mediated hepatoprotection. First, we derived an antisense peptide to the central region of α-MSH that serves as the pharmacophore for melanocortin receptors by the transcription of complementary mRNA sequence of α-MSH in 3’→5’ direction (Scheme 1) [1,2,3,4,5,6]. Second, tryptophan spectro-fluorometric titration was applied to evaluate *in vitro* binding of α-MSH to the antisense peptide. Third, we showed that antisense peptide abolishes hepatoprotective effects of α-MSH *in vivo*.

## 2. Results and Discussion

### 2.1. Molecular Recognition of Amino Acids and Related Antisense Peptides

The Standard Genetic Code defines nucleotide patterns that code for individual amino acids and their complementary, *i.e.*, antisense, pairs [1,2,3,4,5,6,7,8]. The genetic code has 64 codons consisting of three nucleotide bases, each triplet coding for one amino acid [1,2,3,4,5,6,7,8]. Sixty one of them code 20 amino acids and three are stop signals for the protein synthesis. Genetic coding of specific, possibly interacting, amino acids was first discussed by Mekler, Idlis and Biro [2,3]. Root-Berstein, Blalock and others investigated applications and evaluation of the complementary peptide-receptor interactions [1,2,3,4,5,6,7,8,9,10,11,17,18,19,11,17].

The molecular recognition procedure based on the genetic code patterns uses sense-antisense peptide pairs constructed from the complementary mRNA sequences transcribed in either 3’→5’ (left to right) or 5’→3’ (right to left) direction [1,2,3,4,5,6,7,8,9,10,11,17,18,19,20,11,17], as illustrated in Scheme 1 and Table 1. During this process four nucleotide bases are used: uracil (u) is transcribed into its complement adenine (a) and cytosine (c) is transcribed into complement guanine (g), or *vice versa*.

**Table 1:** Amino acids and their antisense pairs obtained from the genetic code [1,5].

Amino acid	Codons for amino acids	Kyte-Doolittle hydropathy scale		Antisense	
		subgroup	value	3'→5'	5'→3'
R (arginine)	cgc, cga, cgg, cgu, aga, agg	polar	−4.5	A, S*	A, S*, P*, T*
K (lysine)	aaa, aag	polar	−3.9	F	F, L
Q (glutamine)	caa, cag	polar	−3.5	V	L
N (asparagine)	aac, aau	polar	−3.5	L	I, V
E (glutamic acid)	gag, gaa	polar	−3.5	L	L, F
D (aspartic acid)	gac, gau	polar	−3.5	L	I, V
H (histidine)	cac, cau	polar	−3.2	V	V, M
P (proline)	ccc, cca, ccu, ccg	neutral	−1.6	G	G, W, R*
Y (tyrosine)	uac, uau	neutral	−1.3	M*, I*	I*, V*
W (tryptophan)	ugg	neutral	−0.9	T	P
S (serine)	ucg, uca, agc, agu, ucu, ucc	neutral	−0.8	S, R*	G, R*, T, A*
T (threonine)	aca, acg, acc	neutral	−0.7	W, C*	G, S, C*, R*
G (glycine)	ggg, ggu, gga, ggc	neutral	−0.4	P	P, S, T, A*
A (alanine)	gcg, gcu, gcc, gca	nonpolar	1.8	R	R, G*, S*, C*
M (methionine)	aug	nonpolar	1.9	Y*	H
C (cysteine)	ugu, ugc	nonpolar	2.5	T*	T*, A*
F (phenylalanine)	uuu, uuc	nonpolar	2.8	K	K, E
L (leucine)	uug, uua, cuc, cuu, cug, cua	nonpolar	3.8	D, E, N	E, Q, K
V (valine)	guu, guc, gug, gua	nonpolar	4.2	H, Q	H, D, N, Y*
I (isoleucine)	aua	nonpolar	4.5	Y*	N, D, Y*

* Deviations from polarity patterns in Molecular Recognition Theory.

**Figure 1:**
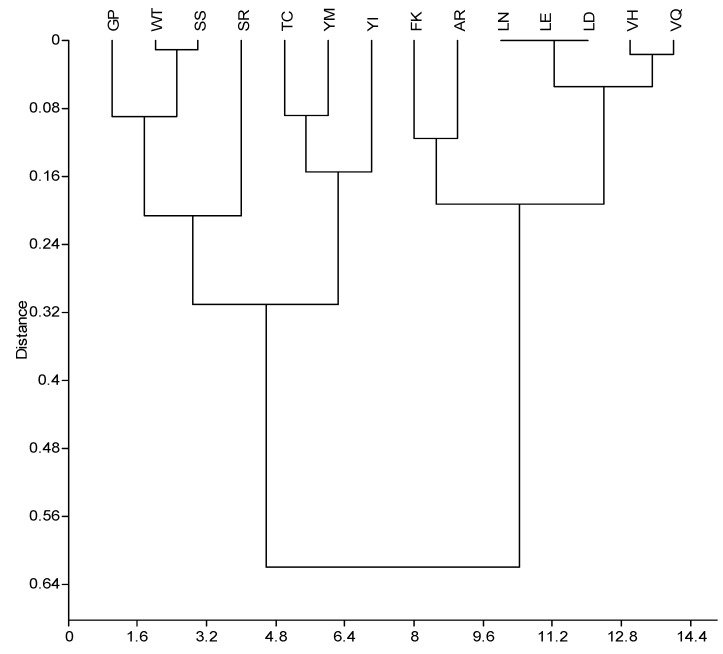
Clustering of complementary amino acid pairs by means of Kyte & Doolittle hydropaty values (3’→5’ direction). Paired group algorithm with Gower similarity measure reveals a strong correlation coefficient of 0.878 [23].

Amino acid pairs arising from this genetic code feature are given in Table 1. Complementary codons within the genetic code define in most cases opposite patterns of hydrophilic and hydrophobic amino acids (Table 1 and Figure 1) [1,2,3,4,5,6,7,8,9,10,11,20]. Uracil is the middle base for most hydrophobic (nonpolar) amino acids and adenine is the middle base for most hydrophilic (polar) amino acids, and as a result of this code property sense and antisense peptides have mutually complementary hydropathy patterns, which according to Blalock *et al.* [1,2,3,4,5,6,7,8,9,10,11,20] may result in their interaction. The number of possibly interacting sense-antisense amino acid pairs within complementary peptides depends on the direction of the sequence transcription. A total number of 27 possible antisense amino acid pairs arises when the nucleotide sequence is transcribed in 3’→5’ direction and 52 antisense pairs are found if it is transcribed in the 5’→3’ direction (Table 1) [1,5,17,18,19,20,21,22]. For practical purposes it is more convenient to model binding of different short antisense peptide motifs by the sequences transcribed into 3’→5’ direction (since there are significantly less antisense peptides per sequence length) [5,17,18,19,20,21,22]. Pentapeptide LVKAT, used as an antisense in this study, was obtained by complementary 3’→5’ sequence transcription of α-MSH (Scheme 1).

Within 3’→5’ arrangement of the complementary amino acid sequences there are 13 amino acid pairs consisting of two different amino acids (e.g., GP/PG, WT/TW, *etc.*) and one self-similar pair of two serines (Figure 1). Small changes of the amino acid molecular polarity influence the secondary protein structure [20], which is relevant for the interaction of sense and antisense peptides [5,10,11]. Blalock *et al.* showed [1,2,3,4,5,6,7,8,11,20,21,22,8,11,20] that in opposite RNA strands hydrophilic and hydrophobic patterns of amino acids are interchanged, while the neutral remain unchanged. Almost 30% (8/27) of the sense-antisense pairs deviations from this rule were observed (Table 1, Figure 1), and this may be the reason why in some cases the patterns of interacting sense-antisense peptides do not follow theoretical assumptions [3,5,10,11].

With respect to the theoretical biology issues complementary 3’→5’ readings of the messenger RNA are more convenient since they correspond to the patterns of transfer RNA anticodons used during the natural peptide synthesis on the ribosome, *i.e.* during translation process [5]. 3’→5’ arrangement of amino acid pairings related to the genetic coding of the protein structure has been observed by Root-Bernstien, and assumed to be possible on a parallel β ribbon [17,18,19]. At this time it is not clear whether the molecular structure of antisense peptide arising from the transcription in 3’→5’ or 5’→3’ direction favors the binding to its sense peptide ligand [3,5,11]. Currently there are no applicable models that link affinity measurement of the sense-antisense peptide binding to 2D and 3D structures of the molecules or the complex [5,22]. However, many examples of successful complementary peptide interactions have emerged from the transcription design in both directions and several physiologically important ligand-receptor systems have been shown to follow predicted amino acid binding patterns [1,2,3,4,5,6,7,8,9,10,11,17,18,19,20,21,22,11,17].

### 2.2. Tryptophan Fluorescence Reveals Sense-Antisense Peptide Interaction

Tryptophan fluorescence was used to detect binding of antisense pentapeptide LVKAT to the central binding region of α-MSH molecule. All spectra in fluorescence titrations were analyzed with the Specfit software [24,25,26,27] and only two spectrally active species were suggested by singular value decomposition (SVD) statistical analysis. One was attributed to α-MSH and the other to its complex with antisense pentapeptide LVKAT. This analysis also suggested 1 to 1 complex formation and did not indicate any higher order complexes. Consequently, the proposed model is given by Equation (1) and Equation (2), where *K*_d_ is the dissociation constant of the complex:

SENSE - LVKAT ⇄ SENSE + LVKAT(1)

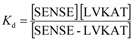
(2)

The dissociation constant for the complex equilibrium of α-MSH with LVKAT was 7.9 ± 0.9 mM. The binding constant of α-MSH to LVKAT is relatively high and implies significant binding affinity of the two peptides. We also investigated binding of the central part of α-MSH sequence (EHFRW) used to derive LVKAT antisense (Scheme 1) and the dissociation constant was 0.20 ± 0.02 mM. These results indicate stronger binding of EHFRW to LVKAT than α-MSH to LVKAT, which can be expected due to the considerably longer peptide chain of α-MSH and consequently sterical and dynamic blocking of binding to LVKAT.

Fluorescence titration was performed with D-α-MSH enantiomer also, but it did not give satisfactory results in terms of the obtained data suggesting very weak (if any) binding under the same experimental conditions. The result of the fluorescence titration of D-α-MSH with LVKAT antisense peptide is consistent with the findings of Turčić *et al.* [15,16] showing that D-α-MSH does not bind antibody to L-α-MSH.

**Figure 2:**
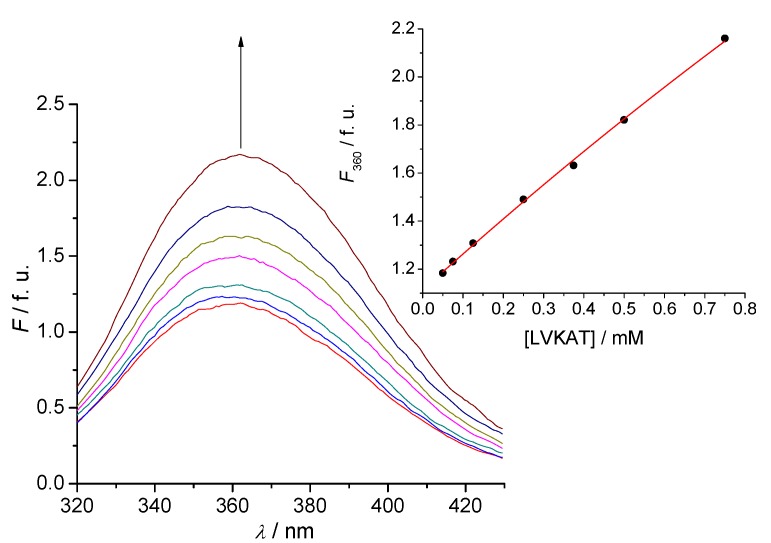
A typical titration of 2.5 µM solution of α-MSH (sense) with LVKAT (antisense) at 25 °C, pH = 7.4, 10 mM phosphate buffer. The concentration of LVKAT was varied from 50 to 750 μM. Fluorescence units (f.u.) are given as a ratio of signals obtained from sample and reference PMTs. **Inset:** Fitting curve at 360 nm according to the Equation (1) and Equation (2).

**Figure 3:**
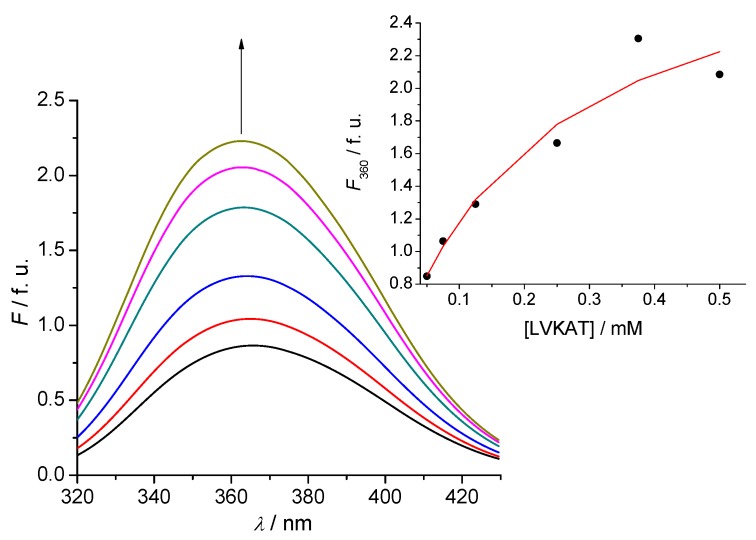
A typical titration of 25 µM solution of EHFRW (sense) with LVKAT (antisense) at 25 °C, pH = 7.4, 10 mM phosphate buffer. The concentration of LVKAT was varied from 50 to 500 µM. Fluorescence units (f.u.) are given as a ratio of signals obtained from sample and reference PMTs. **Inset:** Fitting curve at 360 nm according to the Equation (1) and Equation (2).

Other methods have been also used for the evaluation of complementary peptide interactions. The microtiter plate assay method and high-performance affinity chromatography enabled direct quantitative characterization of peptide recognition [1,28,29]. Electrospray ionization mass spectrometry, NMR spectroscopy, biosensor based surface plasmon resonance and resonant mirror analyses are useful spectroscopy methods to evaluate noncovalent peptide-antisense peptide interactions [3,9,10,19,30,31]. It is, however, worth mentioning that tryptophan fluorescence method that we used for the evaluation of peptide interactions in this study proved to be relatively simple and efficient. With respect to the measurement and experimental settings the interaction of dissolved peptides is within the range of physicochemical parameters (pH, temperature, *etc.*) that resembles physiological situation [15]. The structure of peptides did not significantly affect binding, since short peptides have often undefined structure and observed α-MSH enantiomers exhibit predominantly random coil structure when dissolved in 10 mM phosphate buffer at 25 °C and pH = 7.4 [15]. 

### 2.3. Modulation of α-MSH Hepatoprotection with Antisense Pentapeptide

The hepatoprotective effects of α-MSH were modeled *in vivo*, by means of its antisense pentapeptide. We tested peptides on the experimental model of acetaminophen (APAP)-induced liver lesions in male CBA mice, a useful animal model of hepatitis often used for the screening of hepatoprotective drugs [32,33,34]. Turčić *et al.* [15,16] recently showed that α-MSH, a well known melanocortin peptide with anti-inflammatory and cytoprotective properties, exhibits strong and dose dependent hepatoprotective effects in this model.

**Figure 4:**
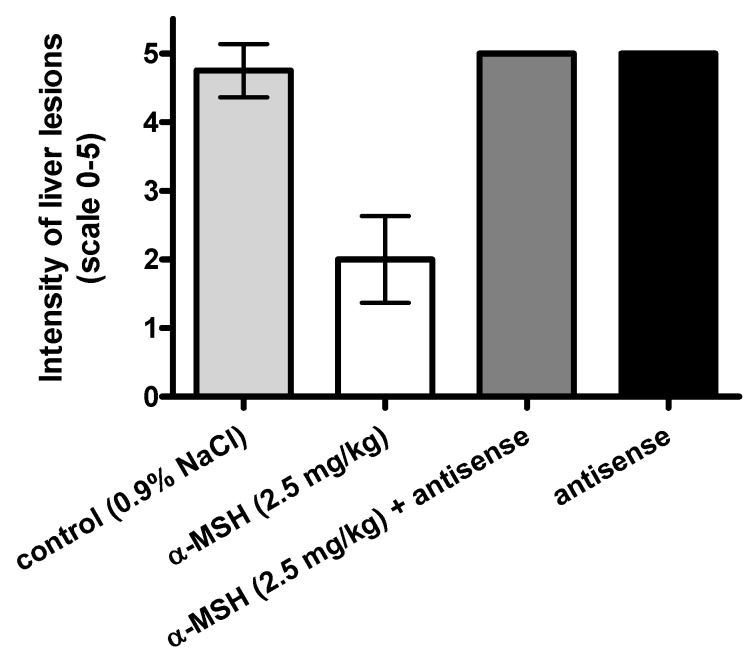
Modulation of α-MSH induced hepatoprotection by means of its antisense pentapeptide LVKAT. Effects of peptides on liver necrosis produced by acetaminophen (150 mg/kg i.g.).

**Figure 5:**
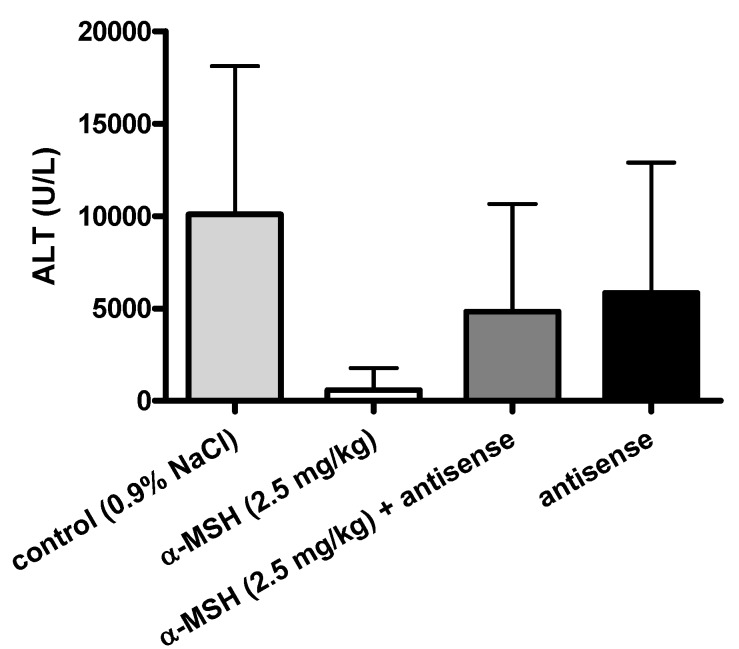
Modulation of α-MSH induced hepatoprotection by means of its antisense penta-peptide LVKAT. Alanine aminotransferase activity (ALT) in plasma of the control and treated animals 24 h after acetaminophen administration.

**Figure 6:**
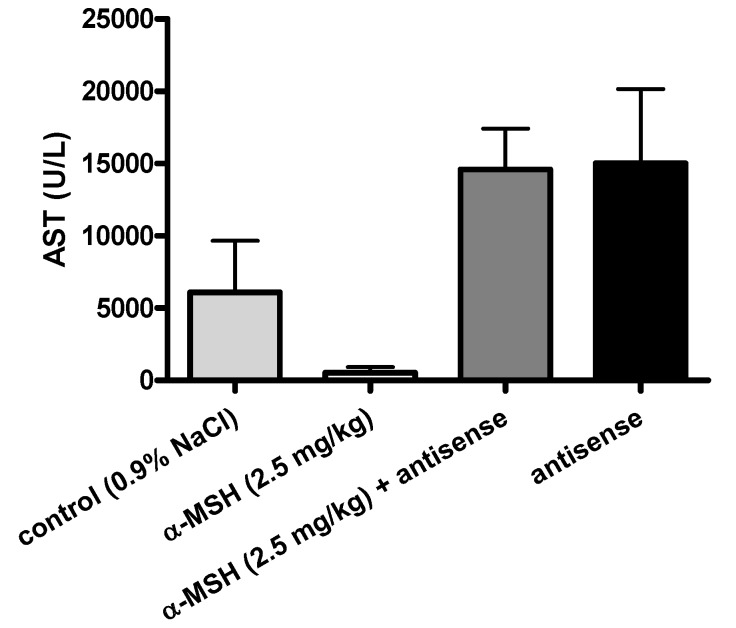
Modulation of α-MSH induced hepatoprotection by means of its antisense pentapeptide LVKAT. Aspartate aminotransferase activity (AST) in plasma of the control and treated animals 24 h after acetaminophen administration.

The effects of α-MSH and its antisense peptide LVKAT on the APAP induced liver lesions (scores on scale 0-5 [15,16,32]) are presented in Figure 4, Figure 5 and Figure 6. Significantly less liver lesions were observed in α-MSH treated animals (2 ± 0.75, mean ± SD), compared to the untreated controls (4.8 ± 0.46, mean ± SD, p < 0.05, Figure 4). The administration of LVKAT antisense together with α-MSH abolished protective effects of α-MSH *in vivo* since the liver lesions of animals treated with equimolar mixture of both peptides (5 ± 0, mean ± SD) did not differ from the untreated controls (p > 0.05, Figure 4). The treatment with antisense peptide was also ineffective (5 ± 0, mean ± SD), which suggested that antisense peptide is not hepatoprotective per se (p > 0.05, Figure 4).

Histopathology findings were confirmed by the measurement of plasma alanine aminotransferase (ALT) and aspartate aminotransferase (AST) presented in Figure 5 and Figure 6, respectively.

We observed significantly lower levels of blood AST (539.5 ± 459.6 U/L, mean ± SD) and ALT (585.8 ± 1424 U/L, mean ± SD) in α-MSH treated animals, compared to the untreated controls (6091 ± 4275 U/L AST, 10087 ± 9595 U/L ALT, mean ± SD), which confirmed the hepatoprotective effects of α-MSH. The administration of LVKAT antisense abolished protective effects of α-MSH *in vivo* since the blood levels of AST (14595 ± 2704 U/L, mean ± SD) and ALT (4827 ± 5555 U/L, mean ± SD) in animals treated with equimolar mixture of peptides did not differ from the AST and ALT in untreated controls (p > 0.05, Figure 5 and Figure 6). The treatment with antisense peptide LVKAT was also ineffective (15040 ± 3217 U/L AST, 5843 ± 4428 U/L ALT, mean ± SD), which confirmed the results of histopathology stating that antisense peptide is not hepatoprotective (p > 0.05, Figure 5 and Figure 6). Blood levels of AST and ALT levels are, together with the histopathology evaluation of liver lesions, standard markers of hepatic damage in experimental hepatitis [15,16,33,34]. Histopathologic evaluation of lesions is a gold standard for the *in vivo* evaluation of the hepatoprotection, and the levels of blood AST and ALT are essential enzymatic blood markers of the liver damage [15,16,32,33,34]. Consequently, our results strongly indicate that antisense peptide LVKAT abolishes hepatoprotective effects of α-MSH on the APAP induced hepatic lesions. 

We showed that antisense peptide binds *in vitro* α-MSH and abolishes *in vivo* its biological effects. α-MSH peptide represents the first 13 amino acids of the ACTH 1-24 molecule [1,5,12,13,14,15,16]. Our *in vitro* results of pentapeptide LVKAT binding to α-MSH obtained by means of tryptophan fluorescence titration are in line with the findings reported by Blalock and Bost for ACTH 1-24 by means of the solid phase binding assay with ^125^I-ACTH [1]. Additionally, we showed that antisense peptide LVKAT successfully blocks hepatoprotective effects of α-MSH *in vivo*. Our results indicate that antisense peptides directed to the receptor binding region of the peptide hormone, *i.e.*, to the functionally important part of the molecule, could modulate its function and abolish its protective effects.

The biological modulation and/or neutralization of sense peptide effects by means of antisense peptides may arise from: (1) peptides binding into molecular complexes (leaving none or low levels of sense peptide to elicit its expected biological effects); (2) partial antagonization of the sense peptide receptor by means of sense-antisense complex; (3) combination of the first two factors; (4) other biological effects of antisense peptide that may not be explained by the involvement of sense peptide and its receptors (e.g. generation of bioactive antibodies to peptides and/or their complexes) [5].

Antisense peptide-based molecular recognition is a useful heuristic algorithm for the rational peptide design of the interacting ligand-receptor sequences ranging in length from ≥4 to <30 amino acids [1,2,3,4,5,6,8,9,10,11,17,18,19,20,21,22,28,29,30,31,35,36]. Despite of the large body of experimental data verifying this theoretical concept a straightforward method for an efficient antisense peptide modelling is still missing. Possible applications of antisense peptides in biomedicine are related to the modulation of peptide and hormone biological function, selective immunomodulation, modelling of discontinuous and linear epitopes, modelling of mimotopes, paratopes and antibody mimetics, peptide vaccine development, peptidomimetic and drug design [1,2,3,4,5,6,8,9,10,11,17,18,19,20,21,22,28,29,30,31,35,36]. In order to achieve better efficiency the algorithm of sense-antisense molecular recognition has to be combined to several other procedures: molecular hydropathy analyses, secondary structure prediction methods and protein database search [20,21,22]. The limitation of the combined antisense-hydropathy analyses is in the fact that it cannot explain 3D protein interactions, but it can be a valuable starting point for more complex computational and experimental analyses [22].

## 3. Experimental

### 3.1. Test Compounds

Test peptides were: (1) L- and D-enantiomers of α-MSH (Ac-SYSMEHFRWGKPV-NH2, mw 1664.9, >95% purity; GenScript, Piscataway, NJ, USA); (2) central region of α-MSH that serves as the pharmacophore for melanocortin receptors (EHFRW, mw 773.86, >95% purity; GenScript); and (3) pentapeptide antisense to central region of α-MSH that serves as the pharmacophore for melanocortin receptors (LVKAT, mw 530.67, >95% purity; GenScript).

### 3.2. Tryptophan Fluorescence Experiment

Fluorescence spectra were measured at 25 °C by OLIS RSM 1000F spectrofluorimeter (Olis, Bogart, GA, USA) equipped with thermostatted cell holder. The excitation wavelength was 280 nm and only the sense substances (α-MSH and EHFRW) and their complexes exhibited fluorescence, whereas the antisense substance (LVKAT) did not. Data obtained from the titrations were analyzed with the Specfit software package [24,25,26].

### 3.3. Treatment Regimen (Hepatotoxicity Model)

Experimental animals were male CBA mice, 12-16 weeks old, weighing 20-25 g and bred at the Ruđer Bošković Institute. Experiments were performed according to the ILAR Guide for the Care and Use of Laboratory Animals, Council Directive 86/609/EEC, and Croatian Animal Protection Act (Official Gazette 135/06). The animals were kept in a room with dark-light cycle (12h/12h) and constant temperature (22 ± 1 °C). Hepatotoxicity was induced according to the slightly modified procedure of Guarner *et al.* [15,16,37,38]. To induce hepatic drug-metabolizing enzymes mice were given 0.3 g/L phenobarbitone-sodium (Kemika, Zagreb, Croatia) for 7 days. Mice were fasted overnight with free access to water 24 hours prior to inducing liver damage by acetaminophen. (Krka, Novo Mesto, Slovenia) 150 mg/kg was given intragastrically (i.g.), via a gastric tube, in a volume of 0.5 mL. Mice were re-fed after 4 hours. α-MSH (2.5 mg/kg), antisense peptide LVKAT (0.8 mg/kg) and their equimolar mixture was given intraperitoneally (i.p.) 1 hour before acetaminophen administration, in a volume of 0.2 mL. Control animals were treated with saline (0.9% NaCl). The size of experimental groups was 6-8. Mice that spontaneously died were excluded from histopathological or biochemical analysis. The starting number of animals was eight per experimental group, none of the animals died in control and α-MSH treated groups, two animals died in groups treated with antisense peptide (LVKAT) and equimolar mixture of antisense peptide and α-MSH. However, the difference was not statistically significant.

### 3.4. Histopathological and Transaminase Estimation of Liver Damage

Mice were sacrificed 24 hours after acetaminophen application. Sections of the liver were fixed in 10% phosphate buffered formalin, embedded in paraffin, sectioned at 4 µm, and stained with hematoxilin and eosin. Sections were examined by using light microscope, and grading of the liver lesions was done on 0-5 point scale according to Silva *et al.* [32] (0 = no lesions, 1 = minimal, 2 = mild, 3 = moderate, 4 = marked and 5 = severe lesions). Alanine aminotransferase (ALT) and aspartate aminotransferase (AST) activity was determined from plasma by standard laboratory techniques. Plasma was separated by 5 min centrifugation at 8000 g, and was stored at −20 °C before transaminase activity determination [15,16]. In normal animals ALT values in plasma were 37.7 ± 7.7 U/L (mean ± SD), and AST values were 109.3 ± 25.3 U/L (mean ± SD). Acetaminophen (APAP) produces enormous rise of both aminonotrasferases in the experimental model of hepatotoxicity in male CBA mice and hepatoprotective effect of the tested substance is evaluated by comparing plasma transaminase values and liver lesions in control and substance treated groups [15,16].

### 3.5. Data Analysis

Statistical analysis was made using KyPlot version 4, and graph plotting was done using GraphPad Prism version 5 for Windows [15,16]. Kruskall-Wallis test and Steel’s test were used to test the differences between effects of applied peptide doses and control group (0.9% NaCl). All applied tests were two-tailed. p ≤ 0.05 were considered as statistically significant.

## 4. Conclusions

(1) Transcription of α-MSH sequence in 3’→5’ direction was used to design antisense peptide (LVKAT) to the central region of α-MSH that serves as a pharmacophore for melanocortin 1, 3, 4 and 5 receptors.
(2) Tryptophan fluorescence titration is a simple and efficient method to evaluate the binding of antisense peptide LVKAT to the α-MSH molecule *in vitro*.
(3) Antisense peptide LVKAT abolished hepatoprotective effects of α-MSH *in vivo*.
